# Beyond seizures: SCN8A heterozygous mutation presenting with epilepsy and paroxysmal dyskinesia

**DOI:** 10.1097/MS9.0000000000002978

**Published:** 2024-12-12

**Authors:** Xinjie Zhang, Tao Yu

**Affiliations:** a Department of Pediatric Neurosurgery, West China Second University Hospital, Sichuan University; Key Laboratory of Birth Defects and Related Diseases of Women and Children (Sichuan University), Ministry of Education, Chengdu, Sichuan, China; b Department of Pediatrics, West China Second University Hospital; Key Laboratory of Obstetric & Gynecologic and Pediatric Diseases and Birth Defects of Ministry of Education, Sichuan University, Chengdu, China

A 48-day-old female infant, born at 38 weeks and 5 days of gestation, exhibited an onset of a multifaceted neurological presentation. Her patient’s history, family history, treatment course, and observed outcomes are all negative or unremarkable. The neonate manifested unprovoked afebrile focal seizures evolving into clustered generalized tonic-clonic seizures (GTCS) (Fig. 1a), along with mild bilateral “shivering” (paroxysmal kinesigenic dyskinesia, PKD), occasionally triggered by intense auditory stimuli (Supplemental video. http://links.lww.com/MS9/A715) (Fig.1b). Genetic testing has revealed a novel de novo SCN8A variant (OMIM: 600702; chr12-51765801; NM_014191.4; p.V892A; Autosomal Dominant Inheritance), indicating the presence of infantile convulsions and paroxysmal choreoathetosis (ICCA) syndrome^[1]^. While mutations in the proline-rich transmembrane protein 2 (PRRT2) gene are commonly associated with paroxysmal kinesigenic dyskinesia (PKD) and benign familial infantile seizures (BFIS)^[2]^, identifying PKD in neonates with SCN8A mutations poses notable diagnostic hurdles.

**Figure 1.**
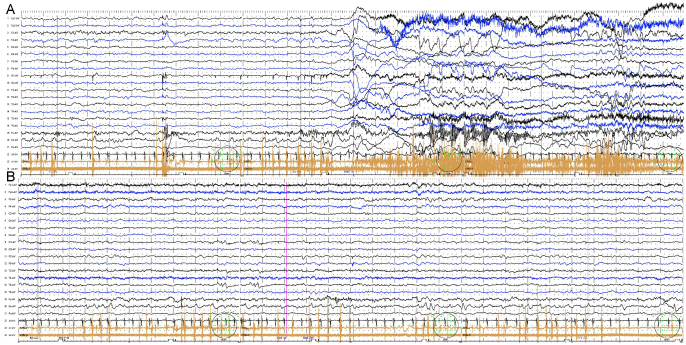
Electroencephalogram (EEG) distinctly illuminates the key disparities between neonatal seizures and dyskinesia, affording profound insights into their characteristic wave patterns. (A) The EEG exhibits low-amplitude rapid activity within the midline frontal lobe, accompanied by conspicuous movement artifacts. Subsequently, rhythmic bursts occur in the bilateral deltoids, evolving into continuous burst activity that is commonly associated with seizures. (B) Conversely, the EEG during dyskinesia events fails to manifest epileptic anomalies. The awake background EEG reveals intermittent rhythmic bursts restricted to the left and bilateral deltoids, which is in line with dyskinesia movements rather than epileptic activity.

The SCN8A gene encodes the voltage-gated sodium channel Nav1.6, which is of crucial significance in neurons as it plays a fundamental role in regulating the generation and propagation of action potentials^[3]^. Mutations in SCN8A lead to abnormal sodium channel function, causing neurological alterations like epilepsy and neurodevelopmental disorders, especially epileptic encephalopathy^[4,5]^. SCN8A mutations can cause excessive sodium channel activation or insufficient inactivation, resulting in neuronal hyperexcitability or abnormal discharges and ultimately epilepsy. Many SCN8A mutations patients display developmental delay (intellectual disability, language, and motor development retardation), motor disorders (abnormal muscle tone, involuntary movements, and ataxia), and behavioral abnormalities (attention deficits, hyperactivity, and autism spectrum disorders)^[5]^. In some cases, specific mutations may even result in epilepsy-related brain atrophy or neurodegenerative changes. Furthermore, PKD resembles seizures and is prone to misdiagnosis as an epileptic episode^[6]^, though some patients have intellectual disabilities or movement disorders without epilepsy^[5]^.

At present, unfortunately, there is no specific drug available for epilepsy caused by SCN8A mutations. Nevertheless, the primary treatment goal is to control neuronal hyperexcitability, with the aim of reducing epileptic seizures and thereby improving the patient’s quality of life.

## Treatment for gain-of-function SCN8A mutations

Gain-of-function mutations lead to excessive activation of sodium channels and abnormal neuronal excitability. Sodium channel blockers such as Lamotrigine, Phenytoin, Carbamazepine, and Lacosamide^[7]^. These drugs control epileptic seizures by blocking sodium channel function and reducing abnormal neuronal discharges.Clobazam, a benzodiazepine drug, mainly reduces neuronal excitability by enhancing the inhibitory effect of GABA receptors^[8]^.Flecainide, a relatively new antiepileptic drug, has shown good efficacy in some SCN8A mutation patients^[9]^.

## Treatment for loss-of-function SCN8A mutations

Loss-of-function mutations result in decreased sodium channel activity and reduced neuronal excitability. Although such mutations are less common, treatment may require increasing neuronal excitability to compensate for the functional defect: Phenobarbital reduces epileptic seizures by enhancing the inhibitory effect of GABA.

Other excitatory drugs, such as certain drugs targeting other ion channels, are potentially used to improve neural signal propagation. The ketogenic diet may help reduce epileptic seizures in patients who are refractory to drug treatment. Vagus nerve stimulation (VNS) may be an adjunctive treatment for patients with refractory epilepsy^[10]^.Gene therapy (under research): future correction of SCN8A mutations may be possible through gene editing techniques such as CRISPR-Cas9^[11]^.

Due to the diverse types of SCN8A mutations and significant differences in patient manifestations, treatment requires individualized adjustment based on specific mutation types and symptoms. Identifying the type of SCN8A mutation is crucial for selecting an appropriate treatment plan. Epilepsy treatment may require the joint efforts of neurologists, genetic experts, and rehabilitation therapists.

In conclusion, this particular case underscores the significance of thorough genetic and neurophysiological assessment in newborns with intricate seizure disorders to achieve accurate diagnosis and personalized treatment. The treatment of epilepsy caused by SCN8A mutations requires a combination of drugs, diet, and other adjunctive therapies. Future precision treatment targeting mutations, such as gene therapy, may bring better therapeutic effects.

## Data Availability

Available upon reasonable request.
